# Reducing ultraviolet radiation exposure among outdoor workers: State of the evidence and recommendations

**DOI:** 10.1186/1476-069X-6-22

**Published:** 2007-08-08

**Authors:** Karen Glanz, David B Buller, Mona Saraiya

**Affiliations:** 1 Department of Behavioral Sciences and Health Education, Rollins School of Public Health, Emory University, 1518 Clifton Road, NE, Atlanta, Georgia, 30322, USA; 2 Klein Buendel, Golden, Colorado, USA; 3 Division of Cancer Prevention and Control, National Center for Chronic Disease Prevention and Health Promotion, Centers for Disease Control, Atlanta, Georgia, USA

## Abstract

**Objective:**

Outdoor workers have high levels of exposure to ultraviolet radiation and the associated increased risk of skin cancer. This paper describes a review of: 1) descriptive data about outdoor workers' sun exposure and protection and related knowledge, attitudes, and policies and 2) evidence about the effectiveness of skin cancer prevention interventions in outdoor workplaces.

**Data sources:**

Systematic evidence-based review.

**Data synthesis:**

We found variable preventive practices, with men more likely to wear hats and protective clothing and women more likely to use sunscreen. Few data document education and prevention policies.

**Conclusion:**

Reports of interventions to promote sun-safe practices and environments provide encouraging results, but yield insufficient evidence to recommend current strategies as effective. Additional efforts should focus on increasing sun protection policies and education programs in workplaces and evaluating whether they improve the health behavior of outdoor workers.

## Background

Skin cancer is the most common type of cancer in the United States [1]. In 2006, more than one million people were diagnosed as having basal cell carcinoma or squamous cell carcinoma, resulting in approximately 2200 deaths from both cancers combined. Melanoma, the third and most often fatal type of skin cancer, is expected to be diagnosed in approximately 59,940 people and to account for about 8110 deaths in 2007 [2]. Between 1975 and 2004, the annual age-adjusted incidence rate for melanoma (new cases diagnosed per 100,000 people) nearly tripled, from 6.8 to 18.5 cases per 100,000. The rate of deaths attributed to melanoma also increased by about 60%, from 1.6 to 2.6 per 100,000 people [3].

### Risk factors for skin cancer and sun-protective behaviors

High levels of exposure to ultraviolet (UV) radiation increase the risk of all three common forms of skin cancer, and approximately 65%-90% of melanomas are caused by exposure to UV radiation [4]. Other risk factors for skin cancer include having fair skin, hair, and eyes (typically correlated with race/ethnicity, albeit imperfectly); and having many moles or nevi [5]. Behaviors that can reduce skin cancer risk include limiting or minimizing exposure to the sun during midday hours when UV radiation peaks (10 am to 4 pm); wearing protective clothing; and using appropriate sunscreen protection. Although sunscreen is thought to be an important adjunct to other types of UV protection, it should not be counted on to provide UV protection by itself. The evidence is inconclusive about whether sunscreen can help to reduce the incidence of basal cell carcinoma or melanoma [6].

### The outdoor workforce and skin cancer

According to the 1991 US. Census Bureau, more than 8% of the US. national workforce (over 9 million workers) primarily work outdoors [7]. High rates of nonmelanoma (basal cell and squamous cell) skin cancer have been found among occupational groups that work outdoors, and rates for nonmelanoma skin cancer among outdoor workers are significantly associated with cumulative UV exposure [8-10]. Because outdoor workers receive intense and prolonged exposure to the sun and are at increased risk of developing squamous cell cancer, interventions that educate these workers and modify their work environments could provide substantial benefit. Some researchers have suggested that the risk of melanoma falls with a continuous pattern of occupational exposure, a recent meta-analysis found the association to be non-significant [11]. This is a methodologically complex issue: it is important to note that the baseline category of no or little occupational exposure is not a "no sun exposure" category because it may include people with heavy recreational sun exposure. Thus it would be wrong to infer from the available data that occupational sun exposure is protective against melanoma [12].

This paper summarizes the state of knowledge about outdoor workers' sun exposure and sun-protection practices, and describes and updates the methods and findings of a systematic evidence review of the effectiveness of interventions to reduce UV radiation exposure among outdoor workers, in order to prevent skin cancer. The systematic evidence review was conducted for the *Community Preventive Services Guide *[13,14]. Recommendations resulting from the evidence review are put in the context of research and occupational health practice and policy.

### Sun exposure and sun safety behavior among outdoor workers

#### Definition of Outdoor Workers and Study Populations

An issue of particular interest among outdoor workers is work done during midday hours when ultraviolet radiation is at its peak. Outdoor workers also may spend more time outdoors during their time off [15], and therefore expose their skin to high doses of ultraviolet radiation. It is common for outdoor workers to spend many years in their occupations [16,17], so their exposure to intense UV rays occurs throughout their lives.

While the outdoor workforce is comprised of a variety of occupations, only a few occupations have been subjected to careful study regarding sun exposure and sun safety. The most commonly studied occupational groups involved the outdoor recreation industry in the United States and Australia. One unique group studied in this industry are workers in the North American ski industry such as ski instructors, ski patrollers, maintenance crews, and base operations who are exposed to the sun during the winter months when UV rays are relatively low but still sufficient at high elevations to achieve substantial exposure [18,19]. Other occupations studied include farmers in the United States and Canada; outdoor utility company employees in Israel and Australia; and bay fishermen (known as "watermen") on the mid-Atlantic east coast of the United States [20]. One study explored the sun exposure of construction workers, transportation workers, and US. Post Office mail carriers [21] and another study observed farmers, construction workers, road workers and other outdoor workers [22], allowing for comparisons between occupations. Finally, a few studies have examined samples that include workers in a variety of outdoor occupations, usually by sampling from general populations of residents [23-25].

A substantial number of outdoor workers have fair skin types that are at high risk for skin damage – Types I (always burns) and II (sometimes burns) [21,23] – placing them at additional risk for skin damage and skin cancer, with their high rates of sun exposure. It is unclear whether risk factors for skin cancer motivate workers to take preventive steps. Two studies found that such risk factors were not associated with greater sun safety practices [21,23]. A prior diagnosis of skin cancer may motivate outdoor workers to take more precautions against sun exposure but even these groups may not limit their time in the sun [15,26].

Differences in sun-related behavior within and between occupations may reflect in part the sun-related habits of the local culture. For instance, the gender composition of study samples differs considerably across outside occupations. Females are more prevalent in studies of aquatic occupations and recreational or day camp staff [27-29], while males predominate in farming, utility, construction, transportation, postal, and ski industry occupations [16,18,21,30-32]. Sun-safety habits of outdoor workers mirror common gender norms for sun safety and may also be influenced by socioeconomic conditions. Thus, the observed patterns of solar protection by outdoor workers need to be interpreted within the context of other population characteristics.

#### Sun exposure of outdoor workers

Studies reported in the literature that provide descriptive information on the sun exposure and sun protection habits of outdoor workers are summarized in Table 1. The studies on outdoor workers demonstrated that they experience a substantial amount of sun exposure on a daily basis. In a national population survey of residents in Canada, respondents who worked outdoors reported receiving on average two or more hours of sun exposure per day [25]. Half of the outdoor workers interviewed in Malta said they worked in the sun for more than 3 hours per day [24]. Likewise, sun exposure of farmers was estimated at 4.15 hours in a survey of Wisconsin dairy farmers [16] and more than 75% of the time spent on the job in a survey of California farmers [32]. A Danish study that used time-stamped personal dosimeter readings found that gardeners received most of their UVR dose on working days [33]. Construction workers, transportation workers, and mail carriers in the United States also spent a large amount of time working outdoors in their jobs (7.9 hours, 7.0 hours, and 5.1 hours per day, respectively) [21].

**Table 1:** Descriptive studies of sun exposure and sun-protective habits among outdoor workers

**Author, Date**	**Population**	**Data Collection Method**	**Sample Size**	**Response Rate**
Bridges et al., 2004	Maryland watermen – whose work is fishing/harvesting crabs, oysters, etc.	Self-administered surveys	63	Unknown
Buller et al., 2003	Ski area employees in the United States and Canada	Self-administered surveys	7,289	Unknown
Garbe & Buettner, 2000	General outdoor workers – control cases from Germany, Austria, and Switzerland in a case-control dermatology study	In-clinic interviews	498	Not reported
Ing et al., 2002	Farmers in Ontario, Canada	Focus group discussions	34	Not reported
Marlenga, 1995	Male dairy farmers in Wisconsin, USA	Self-administered mail survey	202	38%
Moehrle et al., 2003	Mountain guides in Europe	Dosimeter assessment of UV radiation exposure	9	Inapplicable (not a sample study)
Parrott et al., 1996	Farmers, construction workers, road workers, and other outdoor workers in Georgia, USA	Intercept survey, field observations, and in-depth interviews	**Survey**: 155 farmers **Observations**: 49 farmers, 41 construction workers, 39 road workers, 15 other outdoor workers **In-depth interviews**: 9 farmers	Not reported
Rigel et al., 1995	Ski instructors in Colorado USA	UV dosimeters	10	Not reported
Rosenman et al., 1995	Farmers, ≥ 40 years of age, and their spouses in Michigan, USA	Self-administered mail survey	1,342	64%
Scerri et al., 1995	General outdoor workers in a sample of pedestrians in Malta	Intercept survey	559	97%
Schenker et al., 2002	Farmers in California, USA	Telephone survey	1,947	80%
Shoveller et al., 2000	General outdoor workers, Canada national sample	Telephone survey	4,023 adults in entire sample; 546 were outdoor workers	69%
Stepanski & Mayer, 1998	Construction workers, transportation workers, and mail carriers in California, USA	Field observation and self-administered survey	**Observations**: 140 construction workers; 102 transportation workers; 106 mail carriers **Survey**: 63 construction workers; 55 transportation workers; 122 mail carriers	**Observations**: Not reported **Survey**: 73%
Woolley et al., 2002, 2004	General outdoor workers in a sample of men with a previous diagnosis of nonmelanoma skin cancer in Queensland, Australia	Self-administered mail survey	300	62%

Even employees of the North American ski industry who were tested in the winter had substantial sun exposure. Rigel et al. [19] had ski instructors in Colorado wear personal ultraviolet radiation monitors in November and December. They measured both UV-A and UV-B, two main types of UVR that are thought to cause melanoma and sunburn, respectively. Mean daily UV-B exposure was 62.08 mJ/cm^2 ^with a range from 0.5 to 7.6 minimum erythemal doses (MED; 1 MED is sufficient to produce a sunburn) for a person with type II skin. Ten percent received more than 1 MED/h at peak daily exposure times and hourly midday exposure approached 2.5 MEDs for fairer-skinned employees. Exposure to UV-A averaged 10.6 J/cm^2 ^per day (range = 0.5–28), an average daily exposure of 0.55 minimum melanogenic doses (MMD; range = 0.05–1.4 MMD) for a person with type II skin. Many of the worksites in this industry are at higher elevations and have reflective snow surfaces where UV radiation can be substantial. Not surprisingly, then, 45% of ski area employees reported that they were sunburned in the last 6 months (and 8% received a blistering sunburn) when surveyed [18].

Men with a prior history of nonmelanoma skin cancer represent another unique population because of their increased risk for developing further skin cancers. A survey in Australia confirmed that among this type of population, those who work outdoors spent up to 6 hours in the sun and spent at least two hours in the sun on the average weekend day [15]. These data suggest that many of these men had high lifetime sun exposure and continued to receive large doses of UV radiation daily. One should not be surprised, then, that more of the men who worked outdoors in this sample reported that they had been sunburned since their last skin lesion was excised than men who worked primarily indoors. The high chronic exposure and sunburning may compound their risk for recurrence of skin cancer.

#### Sun protection by outdoor workers

Despite the large amount of daily sun exposure, the studies of outdoor workers showed that some workers are taking precautions but a substantial number still are exposed to the sun without adequate protection. In an observational study, about two-thirds of transportation workers compared to approximately 40% of construction workers and mail carriers were observed to be wearing adequate sun protection as measured by the extent to which various body areas were covered [21]. The between-group differences were likely due to required clothing policies that were enforced by the employers [21]. Surveys of a larger sample of postal workers in Southern California revealed that only about one-quarter of letter carriers wore sunscreen and the same proportion wore a wide-brimmed hat while at work [34]. White workers were more likely to use sunscreen and sunglasses always, but hat-wearing did not vary significantly by ethnoracial group [35].

Observations of farmers, road workers, construction workers and other outdoor workers in Georgia found that almost none (only 5%) were wearing widebrimmed hats or caps with flaps or long-sleeved shirts and 26% wore no eye covering, but most of them (86%) wore long pants [22]. A study of male Latino farmworkers in California found that rates of wearing any hat and log-sleeved shirts were high, but few of the men used sunscreen or wore a wide-brimmed hat [36].

Self-reports by outdoor workers indicated that many go without adequate sun protection and some types of protection strategies are more commonly used then others. In the Central European sample, 53% of outdoor workers said that they usually exposed unprotected body parts to the sun and those who did so reported that they had done so for an average of 23 years [37]. The most frequently reported primary prevention strategies by Canadian outdoor workers were wearing protective clothing (60%) and hats (58%), but sunscreen use was infrequent (23% used it on the face and 18%, on the body) [25]. Hat use (37%) exceeded the use of sunscreen (25%) among residents of Malta, too [24].

The most frequent primary prevention strategy by farmers in Wisconsin, California, and Georgia was wearing long pants and a hat [16,22,32,36]. However, wide-brimmed hats that provide shade to all parts of the head and neck were not as popular as baseball caps that shade only the face. Sunscreen and long-sleeved shirts was used very infrequently by farmers, as well. Similarly, only 30% of construction workers, transportation workers, and mail carriers said they were wearing a sunscreen when surveyed (nearly all of these wore a sunscreen with a Sun Protection Factor of 15 or more) [21]. By contrast, many ski area employees wore sunscreen (63%) and lip balm (65%), and hats (62%) [18].

#### Gender differences in sun protection by outdoor workers

There are clear sex-specific differences in sun protection among several samples of outdoor workers. California farmers showed that primary prevention strategies were more frequent among those who were older, female, smoked less, were unmarried or not cohabitating, and were more concerned about skin problems than the younger, usually married, and unconcerned males [32]. Male farmers in Michigan said that they were less likely to engage in solar protection than females [26]. Male outdoor workers in the Central European and Malta samples were much more likely not to wear sunscreen then their female counterparts [24,37]. These patterns are consistent with protection habits observed in surveys of general populations where sun safety is less normative for men than women, men prefer hats, and women prefer sunscreen [37-40]. Moreover, within the populations of outdoor workers, female farmers reported wearing sunscreen more frequently than male farmers; and fewer women wore long-sleeved shirts than men [32]. An apparent exception to the gender norm was reported in the study of Australian men with a prior history of skin cancer where the majority of workers said that they used sunscreen [15]. However, sunscreen was actually used by fewer men in this sample who worked in high rather than low sun exposure occupations [41], so this sunscreen use may still be somewhat less normative for males despite their personal encounter with skin cancer.

Personal risk for skin damage and skin cancer may stimulate outdoor workers to practice more sun safety; this association may also reflect that older workers are more likely to have direct experience with skin cancer or skin damage. Solar protection was more frequent by men in Australia with a prior history of skin cancer, although as noted earlier many were outdoors for long periods and were developing sunburns. The majority wore a wide-brimmed hat (77%) and sunscreen (72%). However, fewer wore long-sleeved shirts (44%) [15]. Among Michigan farmers and their spouses, men reported that they were more likely to protect their skin if they had a prior history of skin cancer [26]. Contrary to these trends, fairer-skinned outdoor workers in Malta wore sunscreen less often, and the use of primary prevention techniques was unrelated to amount of time workers spent outdoors in the sun [24].

#### Strategies that reduce sun exposure

Prevention strategies that rely on reducing sun exposure by either avoiding being outdoors or working in the shade were less popular practices. In the Canadian survey, only 44% of outdoor workers said that they seek shade and 38% avoid the sun when working. Construction workers, transportation workers, and mail carriers also were not likely to report limiting their time in the sun [21]. Ski area employees reported infrequently limiting their time in the sun or staying in the shade while working [18]. These sun safety techniques may conflict with work procedures that require employees to be outdoors for long periods of time each day. Individual employees may be nearly powerless to alter outdoor work hours without a change in workplace policy or actions by employers to provide more shade in outdoor work areas. This underscores the important role of employers' responsibility to provide for sun protection among their workforce.

#### Secondary prevention strategies by outdoor workers

Finally, secondary prevention of skin cancer through a clinical skin examination by a medical professional also seems infrequent among outdoor workers. In a sample of farmers and their spouses from Michigan, 66% of men and 69% of women had never received a skin examination [26]. A similar low frequency was reported by Georgia farmers [22]. Older age and a college education positively predicted obtaining a clinical skin examination. A greater proportion of more affluent males had received a skin examination than less affluent males. By contrast, clinical skin examinations were reported most frequently by older and more affluent females. Most farmers in Georgia did not know how to conduct skin self-examinations. Unfortunately, with only two studies on secondary prevention, any conclusion about its use by outdoor workers is speculative at best.

#### Sun-related knowledge and attitudes

Several of the studies examined outdoor workers' knowledge and attitudes related to sun exposure, skin cancer, and sun protection; specifically their perceived susceptibility to and severity of skin cancer, the efficacy of secondary prevention, barriers to sun protection behaviors, and self-efficacy beliefs about sun protection. Ontario farmers felt that sun safety was an important but not well recognized health issue among farmers [42]. Fewer than 10% of Michigan farmers and their spouses surveyed felt it was very likely that they would develop skin cancer [26] but 43% of Wisconsin dairy farmers and 66% of Georgia farmers believed they would get skin cancer [16,22]. Most of the Wisconsin and Georgia farmers also felt they were more likely to get skin cancer than the average person as a result of their occupation. Perceived severity of skin cancer was moderate among the Wisconsin and Georgia farmers. While nearly all felt it was serious, almost as many did not expect it to affect their ability to continue farming [16,22]. It was ranked as a "top five" health problem among farmers, but behind accidents/injuries, stress/depression, arthritis, and lung disease.

Wisconsin, Georgia, and Michigan farmers expressed beliefs that prevention strategies were efficacious. A large majority of Wisconsin and Georgia farmers felt that daily protection and/or typically recommended protection strategies would reduce the risk of skin cancer [16,22]. Likewise, most Michigan farmers and their spouses believed that early detection would increase the chances of skin cancer being cured and decrease how long a person had to worry about skin cancer [26]. Wisconsin and Georgia farmers were well informed about skin cancer with 70% correctly responding to a skin cancer knowledge assessment [16,22].

A few studies assessed self-efficacy beliefs regarding sun safety and found that they were strong in farmers and ski area employees. For instance, many Georgia farmers were confident that they could wear a wide-brimmed hat (73%), sunscreen (63%), and long-sleeved shirt (48%) while working [22]. Most ski area employees (63%) said they were confident that they could protect themselves from the sun [18]. The perception that one can readily take precautions against the sun may explain why it is not viewed as a more severe health issue by outdoor workers.

Several barriers to sun protection were reported by outdoor workers. Canadian outdoor workers and Georgia farmers said that they did not practice sun safety because they forget, it was inconvenient, they wanted to get tan, and/or they were unconcerned about sun exposure [22,25]. The belief that one looks better with a tan was also expressed by just over half of the Wisconsin farmers [16]. Postal workers in California reported that very few received encouragement from either a co-worker or a household member to wear a hat or sunscreen [34]. Further, the most frequently expressed barrier to sun protection was that it was too hot to wear protective clothing such as hats, long-sleeved shirts, long pants, and work gloves. Georgia farmers also expressed concerns that it was too hot to wear protective clothing [22]. These barriers may keep outdoor workers from practices protection despite moderate concerns about skin cancer and strong beliefs that they are capable of taking adequate precautions. Therefore, it is important to consider workplace environments and policies that may be protective despite individual employees' opinions that sun protection may be inconvenient or uncomfortable.

#### Sun-related workplace education and policies

Two studies provided glimpses into the actions of employers regarding sun protection. In the Canadian survey, only 21% of outdoor workers said that their employers were sources of information about sun safety; most reported that they obtained this information from television, magazines, family, and friends [25]. In the Australian sample of men with a prior history of skin cancer, 37% of the men who worked outdoors reported that their workplace did not require them to use sun protection [15], which is unfortunate because this same survey showed that mandatory company policies on sun protection increased men's use of wide-brimmed hats and shirts with long sleeves. The paucity of data on these subjects makes it difficult to draw any conclusions about the actions of employers regarding sun safety for their outdoor workforces, yet it can be said that workplace sun protection policies may represent an effective but unrealized method for promoting primary prevention of skin cancer.

### Evidence review of skin cancer prevention studies in outdoor workers

As part of the *Community Preventive Services Guide *[43], a systematic evidence review of the effectiveness of interventions to reduce exposure to UV radiation among outdoor workers was conducted. The purpose of the review was to determine: how effective are educational or policy approaches oriented to outdoor workers in improving workers' sun protective behaviors, and which approaches are most effective?

The *Community Guide *includes a series of reviews of intervention research on reducing exposure to ultraviolet light completed for the Cancer Chapter. These reviews examined behavioral, educational, policy and environmental strategies for changing behaviors in order to reduce skin cancer risk and improve health. One review focused on interventions in occupational settings. To be included in the review, the interventions in occupational settings to promote sun-protective behaviors among workers were required to use at least one of the following strategies: (1) provision of information to the workers (instruction, education through small media, or both), (2) additional activities intended to change the knowledge, attitudes, beliefs, intentions, or behaviors of workers (i.e., modeling or demonstration), and/or (3) environmental or policy approaches, including provision of sunscreen and shade. The focus of the review was strictly on **prevention**, not on early detection or patient education for skin cancer.

## Methods

Studies were identified for the review by a comprehensive search of three databases (MEDLINE, PsychINFO, CINAHL) for primary reports of interventions, published in English from 1966–2000, that compared outcomes among persons exposed to interventions with persons not exposed or less exposed to the interventions. A systematic review in which 6000+ titles and citations were screened; 159 articles reviewed; and 85 studies included in the skin cancer prevention review [14]. Additional studies published after 2000 were included if they became available through a call for input to active skin cancer prevention researchers.

For the review in occupational settings, 14 reports that met the inclusion criteria were identified. Of those, there were 8 qualifying studies; 6 reports were excluded due to methodological limitations or because they were reporting on already-included studies. The types of workers who were the focus of the included studies included lifeguards [27,44,45], outdoor recreation staff [28,29], outdoor utility company workers [30,31], and various outdoor workers [23].

Following the standard *Guide *methodology [46], each study was evaluated using a standardized abstraction form and was assessed for suitability of study design and threats to validity. Two abstractors evaluated each study and the abstractions were reviewed, and reconciled when necessary, by a multidisciplinary team of scientists.

A conceptual model, or analytic framework, was developed to show the relationship of the interventions to relevant intermediate outcomes (e.g., knowledge, attitudes, intentions regarding sun-protective behaviors), and to behaviors and skin cancer prevention. Outcome data extracted from the studies were aligned with the analytic framework, to answer specific research questions. Key elements of the framework for the review of interventions in occupations settings were: the intervention; increases in employee knowledge, attitudes and intentions to reduce UV exposure or increase solar protection; changes in exposure and protection; reduction of sunburn; and changes in workplace policies and environments to reduce exposure (e.g., limiting exposure during midday, increasing shade, providing sunscreen, etc.). Although none of the studies identified measured decreased incidence of precancer, nevi, or photodamage; or decreased incidence of skin cancer; it was assumed by the review team that the behavioral changes and reduction of sunburn, if found, would lead to lower rates of cancer [14].

### Summary of community guide review

#### Results of the evidence review

Our search identified 8 qualifying reports (summarized in Table 2) that evaluated the effectiveness of interventions to reduce UV exposure in worksites. The reports involved numerous intervention activities and evaluated a variety of outcomes. Most of the interventions used combinations of strategies, as shown in Table 2. Five reports consisted of interventions that provided sun-safety training to workers; and two. involved sun-protection and skin cancer education sessions and skin exams by a physician. Six studies promoted covering-up behaviors; five involved role-modeling by lifeguards or aquatics instructors; one provided sun protection to outdoor workers (sunglasses, brimmed hat, and sunscreen); one used educational brochures designed for men over the age of 45 and a body chart for self-assessment of pigmented lesions to educate male workers about skin cancer; and two. used environmental supports (sunscreen dispensers and shade structures) to promote sun-protective behavior.

**Table 2:** Studies evaluating interventions to improve sun protection by outdoor workers

**Author, Date, Design, Duration, *Study Quality***	**Population and Sample Size**	**Intervention**	**Results: Summary Effect Measures**	**Limitations**
Azizi et al., 2000 Non-randomized trial 20-month follow-up *Fair quality*	N = 144 (67.6% retention rate) Outdoor workers for Israel Water Company 100% male	Comprehensive/partial/minimal sun protection program(3 groups) *Comprehensive *= local safety officer training, education sessions, protective gear *Partial *= health education, protective gear, brochures *Minimal *= health education, brochures, sunscreen	Increase in sunscreen use in all groups, mostly in Comp. & Partial (+150%) Reduced exposure, highest in Comp. group (-25% skin exposed, -31.5% mean daily occupational exposure)	Recall bias for self-report; UVR dose not validated by other measure; low follow-up rate (68%) and differential (41% in minimal intervention group)
Dobbinson et al., 1999 Non-randomized trial Immediate follow-up and comparison to 9 previous years *Fair quality*	N = 263 Lifeguards in Australia 67% male, 52% < 20 years old	SunSmart campaign program for lifeguards; promotion of long-sleeved shirts, wide-brim hats, sunscreen, shade; raising awareness and providing training for youth	Absolute change in: -regular hat use +34% -regular long-sleeved shirt use + 21% -regular sunscreen +12% -use of shelter +15%	Sampling methods differed by groups; self-reported outcome measures; confounders not assessed
Geller et al, 2001 Randomized controlled trial (RCT) 3-month follow-up *Fair quality*	N = 194 (88.2% retention rate) Lifeguards in Hawaii and Massachusetts 68.7% female, 62.5% white Mean age: 20.9 years	Intervention: sun protection education including training module, materials for sun safety education for children, provision of sunscreen at pool, posters/signs, shade structures, incentives Control group: injury prevention program	Sun protection behaviors measured on 4-point scale: increases in wearing shirts, using shade, and composite sun protection (not sig.). Significant improvement in sun protection policies, significant reduction in sunburns	Self-reported outcome measures; no assessment of participants lost to follow-up
Girgis et al., 1994 RCT 1-month follow-up *Fair quality*	N = 142 (77.4% retention rate) Outdoor workers – Australia 98% male Mean age: 40.5 years	Intervention: skin screening by a dermatologist, education session Control group: no-treatment delayed control group	Absolute change + 16% in % with highest level protection (significant) Significant improvement in knowledge, but no significant attitude change	Sampling frame and site selection not described, loss to follow-up
Glanz et al., 2001 RCT 2- and 5-month follow-up *Fair quality*	N = 176 (71.9% retention at T2, 61.4% at T3; final n = 66) Outdoor recreation staff in Hawaii 60.9% female, multiethnic Mean age: 20.9 years	3-arm trial Intervention Group #1: training/education about sun safety and for conducting children's sun safety program Intervention Group #2: Same as Group #1 plus environmental/policy supports, sunscreen provided, signs, shade, and policy consultations Control Group: Delayed program after first (2 mo.) post-test survey	Sun protection habits score: +1 to 4% change over controls Knowledge increase: + 15% over controls Perceived norms increase: + 18% over controls Sun protection policies: +7% increase > controls Improvements in both Treatment groups, not significant #1 vs. #2	Self-report assessments No assessment of non-responders Sampling method not described
Glanz et al., 1998 Pre-/post-test study 1- to 2-month follow-up *Fair quality*	N = 154 Outdoor recreation staff in Hawaii 66.7% female, multiethnic Mean age: 20 years	Staff training, group activities, children's sun safety program, promotion of sun safe environments and policies	Within-group changes: Sun protection habits score: +1.7% Stage of change: + 9.1% Staff knowledge: + 7.5% Staff sun protection norms: + 5.1%	Self-report assessments Sampling method not described
Hanrahan, 1995 RCT 3-month follow-up *Fair quality*	N = 219 (70% retention rate) Industry workers in Australia 100% male Mean age: 54 years	All groups: knowledge questionnaire + self-exam body chart (delivered at varied times) Intervention group: 2 educational brochures, including questions and answers; self-exam body chart at baseline 2 Control groups: one received self-exam body chart at end of intervention period; other received at same time as intervention group	Increased knowledge about melanoma:+12.6% greater than for controls	No information about sampling or response rate Sampling method not described No report of race/ethnicity and SES of study groups
Lombard et al., 1991 Pre-post test study 1-month avg. follow-up *Fair quality*	N – not reported; done at 2 swimming pools with 600 members Lifeguards in Virginia No description of sample	Peer leader modeling by lifeguards, informational posters and fliers, posted feedback & goals, free sunscreen and commitment raffle; intervention lasted average of 25 days/pool	% lifeguards covering up with target behaviors (hat, shirt, sunglasses, shade, zinc oxide): + 160%, + 675%	No description of sample No statistical testing Convenience sample, 2 pools

In the Results column of Table 2, we summarize the changes found in various study endpoints, and the percentage change for different outcomes. Eleven study arms from seven reports examined changes in sun-protective behaviors and UV exposure and one examined incidence of sunburn. Available reports provided insufficient evidence to determine effectiveness of the intervention in increasing the sun-protective behaviors of covering up. or seeking shade, or in decreasing the incidence of sunburn and UV exposure, because of the small number of reports and inconsistent results.

Six arms from five reports examined knowledge; five arms from four reports examined attitudes or beliefs; and three arms from two reports. examined environmental pool policies. Six arms from five reports demonstrated inconsistent effects on knowledge, and five arms from four reports demonstrated inconsistent effects on attitudes or beliefs. Three arms from two reports. examining sun-protection demonstrated desirable effects of the intervention on sun-safety measures and environmental supports (provision of sunscreen dispensers and portable shade structures) at recreational centers and swimming pools.

According to the *Guide *rules of evidence, available reports provide insufficient evidence to determine the effectiveness of interventions in occupational settings because of too few reports and inconsistent evidence.

#### Other issues examined in the Guide evidence review

In addition to examining the effectiveness of interventions to reduce UV exposure, the evidence review uses the current evidence base to assess whether conclusions can be drawn regarding several other issues: applicability; economic efficiency; barriers to implementation; and other positive or negative effects. In this review, evidence about applicability was not assessed for this intervention because effectiveness was not established. Also, economic evidence and evidence about barriers to implementation were not collected, either, because effectiveness was not established.

With regard to other positive or negative effects, reviewed studies did not include information on other potential effects of these interventions. Other positive effects may include reaching populations that might not otherwise be exposed to skin cancer prevention and reducing risk of overexposure to heat. Potential negative effects of interventions may include worker requests for reductions in time spent working outdoors and increased costs to employers that are passed on to consumers (e.g., taxpaying public, utility customers, swimming pool users, etc.).

#### Recently completed intervention research

Two large intervention studies to improve sun protection among outdoor workers were published since the community guide review was completed. They include a large study of ski area employees in North America [18] and a study of mail carriers in Southern California [47,48]. These studies help to strengthen the evidence base because both employed group-randomized pretest-posttest controlled trial designs, innovative interventions, and highly credible outcome measures; they have been included in Table 2 to update the previous data. The study of ski area employees found a reduction in sunburns among intervention site employees [18] and there was evidence of intervention effects on sun protection behaviors into summer months when the seasonal employees in this industry work elsewhere, many at other outdoor occupations [49]. The study of postal workers found both short-term and persistent effects of the intervention on sunscreen use and wide-brim hat use [48]. In addition, both these studies included systematic analyses of implementation and results showed that there were dose-response effects indicating that employees who were most-exposed to the interventions showed greater increases in sun-protection behaviors [18,48].

#### Interpreting conclusions from the evidence review and important research and practice considerations

The available reports provide insufficient evidence to determine the effectiveness of interventions in occupational settings to reduce UV exposure and increase sun-protection behavior because of too few reports and inconsistent evidence. This does not, however, mean that the individual studies did not find positive effects; but rather, that there were too few well-designed studies to conclude that specific types of strategies are effective. These findings need to be considered in the context of the broader field of skin cancer prevention, and in view of what we understand from descriptive studies of outdoor workers' sun-safety knowledge, attitudes, and practices.

Indeed, we should bear in mind that this is a relatively young field with a small literature, and take these findings as the foundation upon which to build a body of evidence that informs occupational medicine and cancer prevention program design. Future studies should include randomized, often group-randomized designs; longer follow-up periods; multiple outcome measures including those that go beyond self-report to include observations and biological assessments on at least a sub-sample of participants; and vigorous attention to reduce attrition [50]. The recently completed studies by Buller [18] and Mayer [48] illustrate several of these improved study features and are excellent models for continual improvement in this scientific area.

#### Research in progress

Two of the authors on the present manuscript are also investigating issues related to the dissemination of sun safety interventions in workplaces – i.e., how employers decide to adopt sun protection education programs and how well they implement them, outside of a highly controlled research trial. Specifically, Glanz and colleagues are examining the diffusion of the *Pool Cool *sun safety program to swimming pools throughout the United States [50] lifeguards and aquatic instructors are both targets of the intervention and intermediaries who serve as role models to children and other poolgoers. This study includes more than 5,000 lifeguards a year for three years. Buller is studying the dissemination of Go Sun Smart to ski areas in North America through the National Ski Areas Association and its membership. This study includes on-site observations at 69 ski areas and surveys with nearly 500 managers on adoption decisions and implementation actions.

## Conclusion

There is considerable room for improvement in occupational sun protection. Some workers take precautions while working outdoors in the sun, but the vast majority of outdoor workers studied in the United States, Canada, and the Mediterranean region – the regions for which there are multiple publications – do not practice adequate or any sun safety. Sun protection may not yet be a priority in most outdoor work environments in these countries. Changes are beginning to occur in American policies, as indicated by the recent provision in California state law to provide lifeguards who get skin cancer with workers' compensation benefits [51]. Several major unions and employers have developed sun protection guidelines and brochures that can serve as models to other workplaces [52]. The situation in Australia may be better, perhaps due to the influence of 20 years of concerted efforts at educating the public about skin cancer prevention [53]. Elsewhere, education of individual employees or adoption of policies to improve sun safety does not yet occur with great frequency. Employees may find it difficult to practice some commonly recommended sun safety techniques such as avoiding being outdoors, using shade, and wearing protective clothing without changes to work conditions and procedures.

For the greatest possible impact, comprehensive workplace sun safety interventions should be aimed at both the outdoor workers and their employers. When considering a comprehensive approach to workplace safety, several issues should be considered: seasonal outdoor workers who may be at higher risk because of little organizing capacity, workers in unions vs. non-unions, workers in Federal agencies, and self-employed workers such as those on small farms. Appeals to employers about the importance of worker safety in the context of risk management might be successful. Employees who work primarily indoors should not be overlooked. Many receive considerable recreational exposure and exposure occurs in a much more intermittent pattern (a risky pattern associated with melanoma development). Workplace communication also can be used to deliver sun protection advice to employees families. These efforts should be carefully evaluated so that other occupational health and cancer prevention experts can be sure the most effective approaches are adopted and used widely, to achieve the greatest public health benefit.

## Abbreviations

MED – Minimal erythemal doses

MMD – Minimum melanogesic doses

UV – Ultraviolet

UVR – Ultraviolet Radiation

UV-A – One of the main types of ultraviolet radiation

UV-B – One of the main types of ultraviolet radiation

## Competing interests

There is no conflict of interest related to financial interests for any of the authors. However, based on the instructions, we have disclosed that Dr. Saraiya is employed at the CDC, which sponsored the work reported in this paper.

## Authors' contributions

KG coordinated the manuscript. MAS and KG participated in the design of the study and performed the data analysis. KG and MAS conceived of the study, and participated in its design and coordination and helped to draft the manuscript. DB completed literature review and drafted two major sections of the manuscript. All authors read and approved the final manuscript.
